# Pediatric sleep disorders are common and costly: how can we help?

**DOI:** 10.1007/s44470-026-00152-1

**Published:** 2026-08-03

**Authors:** Sally L. Davidson Ward

**Affiliations:** Division of Pediatric Pulmonology and Sleep Medicine, Keck School of Medicine, Children’s Hospital Los Angeles, University of Southern California, Los Angeles, CA USA

Health care costs a bundle in the USA, and sleep disorders in children contribute 2.88 billion dollars annually to this total, this is significantly more than children without sleep disorders, according to the article by Carney et al. recently published in the Journal of Clinical Sleep Medicine [1]. Studies completed in Israel in the early 2000s highlighted the impact of pediatric obstructive sleep apnea (OSA) on health care utilization and also documented the reduction in health care expenditures following treatment [2, 3]. This may be true for all pediatric sleep disorders. The indirect costs of sleep disorders are not captured in the study by Carney et al., such as decreased time in school, impaired quality of life, and missed work by parents. Over-the-counter medication expenditures were also not captured. The survey data included early years of the COVID-19 pandemic which may have been a barrier to seeking health care. Despite these limitations, this study emphasizes the importance of recognizing and treating, curing, or managing sleep disorders in individual children to reduce health care expenditures. In parallel, this should optimize quality of life, reduce the burden of illness and the risk of co-morbidities, improve school attendance, and allow parents to work, care for their family, and themselves. We must also strive to support sleep health in society to create an environment that does not enable sleep disorders. Examples include age-appropriate school start times, help for children and parents to manage the conflict between screen time and bedtime, and decreasing the risk of drowsy driving in teens. We must extol the benefits of healthy sleep for all domains of well-being.

The study by Carney and colleagues utilized the Medical Expenditures Panes Survey (2017–2022) administered by the US Agency for Healthcare Research and Quality. The survey includes households and their respective medical providers (clinics, hospitals, home health, and pharmacies) and a sample of insurance plans offered by employers (https://www.ahrq.gov/data/meps.html). Adjustments were made to account for co-variates and co-morbidities that facilitate or impede accessing health care or impact the need, or perceived need for health care, respectively. The use of a national database collected by the government is worth highlighting. The US Department of Health and Human Services (HHS) is promoting open data access in response to the Open, Public, Electronic, and Necessary (OPEN) Government Data Act of 2018. This is outlined in the *Living HHS Open Data Plan*, Version 1.1, 12/22/20225. HHS is creating metadata standards and inventories with a stated goal to “responsibly unleash the power of HHS data, deliver improved services through data, and maximize the nation’s return on its investment in data to benefit all Americans.” This process is in development with a three-year timeline and provides opportunities to further explore the landscape of children’s health, including sleep disorders. Beyond documenting the impacts of pediatric sleep disorders, what are the avenues for reducing the cost to individuals and society and, more importantly, improving health outcomes for children?

## Support research:

Explore the causes and management of pediatric sleep disorders through new and existing data. It is a hopeful sign that the HHS Make America Healthy Again (MAHA) report on children’s well-being mentions sleep health several times, mainly in the context of screen time competing for sleep time. We need interventions for pediatric insomnia, especially for children with special needs such as autism where even evidence-based behavioral interventions can fall short. Stay up to date on activities and opportunities sponsored by the National Center on Sleep Disorders Research by subscribing to the listserv https://list.nih.gov/cgi-bin/wa.exe?A0=SLEEPRFA-L.

## Create access to care:

Educate primary care providers to include sleep health as part of routine pediatric care, beginning in medical school and continuing through residency. Carney et al. report that pediatric residency education on sleep disorders was found to be minimal in an international survey and that this contributes to pediatricians reporting unease with treating sleep disorders in children [1, 4]. The ABP and ACGME are partnering to reduce the length of pediatric fellowships for trainees aiming to be clinician educators—this may increase the pool of applicants, and the extra year of sleep medicine fellowship could become more attractive. The Advancing Innovation in Residency Education (AIRE) initiative allowing pulmonary fellows to train in sleep medicine concurrent with their primary fellowship, and for board certified/eligible physicians in relevant specialties to train parttime as fellows, is to be celebrated as a novel way to increase the number of pediatric sleep medicine specialists, which currently hovers around only 270 nationally. The AASM and ACGME are to be commended. Perhaps it is worth considering using the AIRE program as a model for trainees in other sleep-related fields such as neurology, psychiatry, and otolaryngology.

## Support telehealth:

Alleviating the need to travel reduces costs and inconvenience for families and patients and allows for the expansion of clinical services without the overhead of brick-and-mortar clinics. The AASM describes telehealth as having immediate potential to overcome barriers to high-quality sleep care to “dramatically increase sleep medicine accessibility and clinical efficacy” (https://aasm.org/advocacy/initiatives/telemedicine).

## Improve access to diagnostic testing:

In-laboratory polysomnography is our gold standard, but access is plagued by long wait times. However, data that home sleep apnea testing is applicable to children and teens are accumulating. Clinical practice guidelines from the AASM for this approach will be welcomed when the time is right and should support coverage by both public and private insurance. This will allow pediatric sleep labs to reduce wait times and to focus on evaluating the most complicated clinical scenarios. Wearables for clinical diagnosis, monitoring, and therapy specifically developed for and tested on children and with FDA approval and covered by insurance are also important to improve access to care, rather than embarking on laborious post-market comparisons to clinical gold standards of expensive commercially available devices. These would go hand-in-hand with the development of therapeutic apps to address behavioral sleep concerns that are evidence-based and, if not free, are covered by insurance.

## Improve throughput:

Artificial intelligence technologies that aid in analysis of polysomnographic data should free up time for clinicians to see patients.

## Expand proven therapies:

Hypoglossal nerve stimulation is FDA approved for adolescents with Down syndrome, why not for other conditions where mask positive pressure is not tolerated? Palatal expansion shows promise for relieving OSA—perhaps this should be a covered benefit for children covered by public insurance. Children should be included in clinical trials of pharmacologic therapy for treating sleep disorders. Off label treatment ensures insurance barriers for coverage and understandable skepticism by parents, patients, and providers. Encourage medications for sleep disorders to be included in the Pediatric Trials Network (https://pediatrictrials.org).

## Become an advocate:

Explore the National Sleep Foundation’s “Best Slept Sleep for a Stronger Nation” and the AASM’s “Sleep for a Stronger Nation” and their Community Sleep Health and Public Awareness Grant. Donate to organizations to support lobbying for sleep health. Give Grand Rounds and be a resource for local school boards and other child advocacy groups. Sign up for the Health Datapalooza sponsored annually by AcademyHealth for “leaders from government, industry, academia, and the patient community to explore emerging data applications, evolving regulations, and what’s next for health data” (https://academyhealth.org/Datapalooza2026).

And finally, be proud of your role in pediatric sleep medicine. We help children sleep!

## Data Availability

Not applicable.
